# Niche specialization of reef-building corals in the mesophotic zone: metabolic trade-offs between divergent *Symbiodinium* types

**DOI:** 10.1098/rspb.2010.2321

**Published:** 2010-11-24

**Authors:** Timothy F. Cooper, Karin E. Ulstrup, Sana S. Dandan, Andrew J. Heyward, Michael Kühl, Andrew Muirhead, Rebecca A. O'Leary, Bibi E. F. Ziersen, Madeleine J. H. Van Oppen

**Affiliations:** 1 Australian Institute of Marine Science, UWA Oceans Institute (M096), 35 Stirling Highway, Crawley, Western Australia 6009, Australia; 2 DHI Water and Environment, Level 2, 83 Havelock Street, West Perth, Western Australia 6872, Australia; 3 Marine Biological Laboratory, Department of Biology, University of Copenhagen, Strandpromenaden 5, 3000 Helsingør, Denmark; 4 Plant Functional Biology and Climate Change Cluster, University of Technology Sydney, PO Box 123, Ultimo, New South Wales 2007, Australia; 5 Australian Institute of Marine Science, PMB 3 Townsville MC, Townsville, Queensland 4810, Australia

**Keywords:** photosynthesis, respiration, photo-acclimatization, ecophysiology, zooxanthellae, Indian Ocean

## Abstract

The photobiology of two reef corals and the distribution of associated symbiont types were investigated over a depth gradient of 0–60 m at Scott Reef, Western Australia. *Pachyseris speciosa* hosted mainly the same *Symbiodinium* C type similar to C3 irrespective of sampling depth. By contrast, *Seriatopora hystrix* hosted predominantly *Symbiodinium* type D1a or D1a-like at shallow depths while those in deeper water were dominated by a *Symbiodinium* C type closely related to C1. The photosynthesis/respiration (P/R) ratio increased consistently with depth at the two sampling times (November 2008 and April 2009) for *P. speciosa* and in November 2008 only for *S. hystrix*, suggesting a reduction in metabolic energy expended for every unit of energy obtained from photosynthesis. However, in April 2009, shallow colonies of *S. hystrix* exhibited decreased P/R ratios down to depths of approximately 23 m, below which the ratio increased towards the maximum depth sampled. This pattern was mirrored by changes in tissue biomass determined as total protein content. The depth of change in the direction of the P/R ratio correlated with a shift from *Symbiodinium* D to C-dominated colonies. We conclude that while photobiological flexibility is vital for persistence in contrasting light regimes, a shift in *Symbiodinium* type may also confer a functional advantage albeit at a metabolic cost with increased depth.

## 1. Introduction

The success of reef-building corals is dependent on their obligate association with symbiotic photosynthetic dinoflagellates of the genus *Symbiodinium*. The ecophysiological characteristics of the symbionts [1] and their coral host [2,3] influence the physiological tolerances of reef-building corals to fluctuating environmental conditions. While adaptation and/or acclimatization of the symbionts to anthropogenic-related changes in sea water temperatures and irradiance in shallow coral reef habitats are well known [4], recent attention has focused on understanding biological processes associated with deep coral reefs of the mesophotic zone (i.e. defined as coral reef habitat from 30 to 150 m; [5–7]). Within the mesophotic zone, downward irradiance decreases exponentially, changing by several orders of magnitude with smaller changes in sea water temperature [5]. Coral species that thrive in such contrasting light regimes must invoke efficient and flexible strategies of photo-acclimatization by the holobiont (e.g. [8–10]). In addition, mesophotic coral communities are considered to have strong functional links to their shallow-water counterparts, potentially acting as refuges and a source of new recruits following disturbances [5,6]. However, an understanding of the metabolic processes associated with the ecological function of corals in the mesophotic zone is needed to better understand their role in the resilience of coral reefs to changes in environmental conditions.

The discovery of high genetic diversity in the genus *Symbiodinium* [11] associated with some corals has highlighted the role of symbionts in niche diversification on coral reefs [12–14]. The genus *Symbiodinium* comprises nine clades (A–I) that are each further divided into multiple types [15,16]. Some of these are known to differ physiologically, which may confer ecological advantage in traits such as growth and thermal tolerance for corals associated with certain *Symbiodinium* types [17–19]. Moreover, the zonation of genetically distinct *Symbiodinium* in some corals along depth gradients has been related to differential photo-acclimatization among *Symbiodinium* types to contrasting light regimes [13,20,21]. Using *in situ* chlorophyll fluorescence techniques, Frade *et al.* [22] found that the light use efficiency as well as symbiont density and a change in symbiont type were important explanatory variables of photo-acclimatization of *Madracis* spp. over a 5–40 m depth range at Curaçao in the Caribbean. Similarly, Lesser *et al*. [7] showed that changes in the maximum quantum yield (*Fv*/*Fm*) and photosynthetic pigments were important in photo-acclimatization of *Montastraea cavernosa* to lowered irradiances at mesophotic depths (3–91 m). While these studies highlight effective capacities of reef-building corals to acclimatize to distinct light regimes, our understanding of metabolic processes of corals in the mesophotic zone remains limited.

Photosynthesis–irradiance (P–E) relationships are often measured in studies of symbiont photobiology as well as the respiration of the holobiont in relation to the optical properties of the water column (e.g. [23]). Symbionts acclimatized to low irradiance are typically characterized by a low maximum photosynthetic rate (*P*_max_), a low minimum saturating irradiance (*E*_k_) and a high light use coefficient (*α*) [8,23]. The reverse commonly occurs under high irradiances owing to protective mechanisms that limit chronic photo-inhibition of the photosynthetic apparatus [24,25]. Other photo-acclimatory responses, such as increased symbiont cell density and/or concentration of chlorophyll, have been observed in corals exposed to low-irradiance regimes. Such corals increase their capacity to capture light for photosynthesis in order to meet their energetic requirements [8,9,26].

The photo-biology and depth distribution of mesophotic coral–algal symbioses in combination are relatively unexplored (but see [7,20,22]). There is a critical need to obtain further information on the ecology of mesophotic coral communities in order to improve our understanding of their role as refugia for shallow-water coral reef communities in an era of rapid environmental change. Here, the influence of *Symbiodinium* communities on photobiology was examined using light saturation (P–E) curves determined from direct oxygen production and consumption of corals incorporating mesophotic depths (0–60 m) at South Scott Reef on Australia's Northwest Shelf.

## 2. Material and methods

### (a) Study area and sampling design

**Figure 1.**
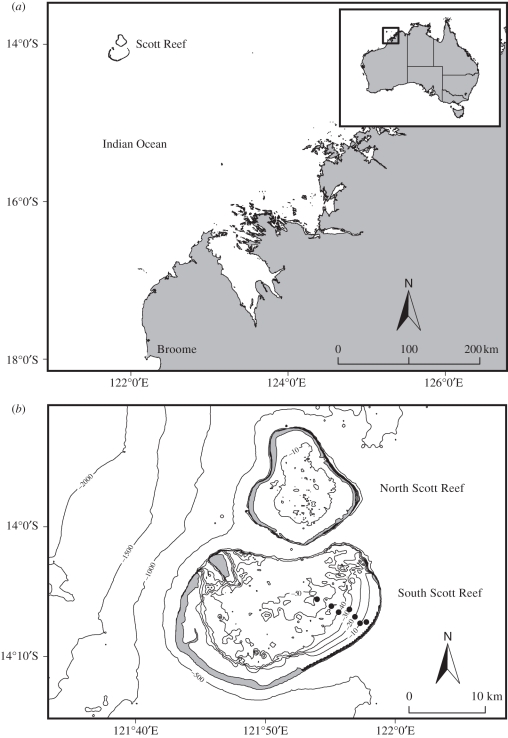
(*a*) Map of Scott Reef on the Northwest Shelf of Australia where a small remotely operated vehicle (ROV) and video benthic grab were used for sampling of reef-building corals over a large (0–60 m) depth gradient. (*b*) Sampling sites for genetic and physiological measurements (circles) at South Scott Reef.

Scott Reef is an isolated coral reef system situated approximately 270 km from the northwest Australian mainland (figure 1*a*) and comprises two coral reefs: South Scott Reef and North Scott Reef (figure 1*b*). It is an offshore coral reef setting with low exposure to anthropogenic stressors. Covering an area of approximately 610 km^2^, the Scott Reef system rises from surrounding water depths of approximately 400 m. A significant proportion of South Scott Reef (approx. 275 km^2^) occurs as a deep lagoon beyond depths of 30 m. Within this, reef-building corals are known to occur as an important component of the benthos from 0 to approximately 65 m depths.

In order to examine patterns of photo-acclimatization, the foliaceous coral *Pachyseris speciosa* (Dana 1846) and the branching coral *Seriatopora hystrix* (Dana 1846) were sampled twice (November 2008 and April 2009) over their entire depth range inside South Scott Reef lagoon, using a combination of a remotely operated vehicle (ROV; LBV 150, Seabotix, San Diego, USA) and a video-mounted van Veen benthic grab. The sampling design for *P. speciosa* comprised the collection of five replicate colonies (*n* = 5) from each of five depths (10, 30, 45, 50 and 60 m; figure 1*b*), whereas five replicate colonies of *S. hystrix* were sampled from each of six depths (3, 10, 20, 30, 40 and 55 m; figure 1*b*). All corals were held in shaded flow-through tanks on-board a research vessel pending photobiological measurements, which were carried out within 1 h of collection.

### (b) Sea water temperature and irradiance

Sea water temperature was measured using a conductivity-temperature-depth (CTD) instrument (SBE 19plus V2 SEACAT; Sea-Bird Electronics, Inc, Washington, USA). Two replicate CTD casts were done at each sampling station in the South Scott Reef lagoon. Maximal quantum scalar irradiance (PAR, 400–700 nm) was characterized at midday at each location using an underwater spherical quantum sensor (LI-193, LI-COR, Nebraska, USA). The light sensor was lowered through the water column and irradiance was measured just below the surface, then at 1 m increments, to a maximal depth of 50 m. The exponential decrease in irradiance with depth was described using Beer–Lambert's Law *E*_*z*_ = *E*_o(*z*)_ e^−*K* d.*z*^, where *E*_*z*_ is scalar irradiance at a given depth, *E*_o(*z*)_ is the scalar irradiance just beneath the surface, *z* is depth in metres and *K*_d_(PAR) is the diffuse attenuation coefficient of scalar irradiance (in units of m^−1^).

### (c) Symbiont type

The genetic identity of *Symbiodinium* communities was examined in both coral species. One of the coral fragments from each colony used for photobiological measurements (*n* = 5, described below) was fixed in absolute ethanol for downstream DNA extraction. Following DNA extraction, zooxanthellae were genetically identified using single-stranded conformation polymorphism (SSCP) methodology targeting the zooxanthella internal transcribed spacer region (ITS1) as described by Van Oppen *et al*. [27] and Fabricius *et al*. [28]. Most samples exhibited SSCP profiles with multiple bands, some of which did not align with our SSCP reference samples of known ITS1 sequence. For representatives of each SSCP profile, the zooxanthella ITS1 PCR product was cloned and all cloned bands visible in the original profile were sequenced in order to identify all sequence types present. Standard cloning and sequencing methods were used [28].

### (d) Photobiology

Fragments of the five sampled colonies of each species and depth were used to examine rates of gross photosynthesis and holobiont dark respiration. For each replicate, a small coral nubbin was attached with epoxy to a glass tile and mounted inside a gas-tight glass chamber (volume 260 ml) filled with filtered (1 µm) sea water. Care was taken to not introduce air bubbles. We ensured that there was no build-up of O_2_ gradients within the glass chamber by stirring with a glass-coated magnetic stir-bar with a magnetic stirrer (2MAG, Munich, Germany). To buffer against changes in sea water temperatures in the laboratory, the chambers were held within a flow-through water bath (100 l) at a controlled flow velocity of 500 ml min^−1^. A planar optode spot (diameter 0.5 cm; Presens, Germany) was attached to the inside of each chamber and calibrated at experimental temperature and salinity from readings in air-saturated and O_2_-free sea water using an optical fibre held in place above the planar optode and connected to either a Fibox3 or OXY-4 mini oxygen meter (Presens, Germany). A 150 W metal halide lamp (HIS-TD CoralArc 20K, Sylvania Lighting, Belgium) attached to a large retort stand was used to provide illumination at nine irradiance steps between 0 and 400 µmol photons m^−2^ s^−1^ as measured using an underwater spherical quantum sensor. The duration of incubation at each irradiance level was 15 min, which was found to be sufficient to obtain a linear change in the O_2_ concentration necessary for robust estimation of O_2_ production and consumption. Minimum saturating irradiance (*E*_k_) of all experimental samples was well within the illumination range and saturation of photosynthesis was observed at the highest irradiances.

**Figure 2.**
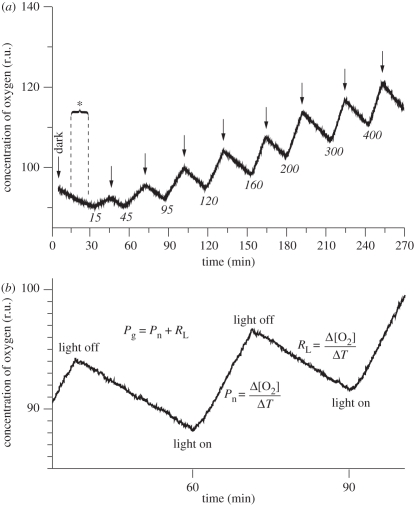
(*a*) Schematic of the measuring sequence of net photosynthesis (*P*_n_) rate and post-illumination respiration rate (*R*_L_) at eight incremental light levels (shown in italics, µmol photons m^−2^ s^−1^). Downward arrows indicate light off following each irradiance treatment. Steady state levels of oxygen were obtained within 15 min of each light or dark treatment. Asterisk (*) denotes initial measure of dark-adapted respiration (*R*_D_). (*b*) Diagram of the calculation of *R*_L_ in the dark and *P*_n_ in the light. Rate of gross photosynthesis (*P*_g_) for each light level was calculated as the sum of *P*_n_ and *R*_L_ measured for that light level.

Net O_2_ production by the zooxanthellae and O_2_ consumption in the dark (respiration) by the holobiont were measured as changes in O_2_ content in the chamber volume, where the chamber volume equals the total volume minus the volume displaced by the coral sample. The initial measure of respiration was conducted on shade-adapted corals (figure 2*a*) and was allowed 30 min in the dark for a measure of dark-adapted respiration (*R*_D_). This measurement was normalized to surface area of the sample and not a tissue biomass parameter as both zooxanthellae and the host contributes to respiration.

Dark-adapted respiration measures are often assumed to be representative of respiration under light in O_2_-based photosynthesis studies. However, this assumption is likely to be incorrect as light dose (irradiance and duration) affects the photophysiology and hence metabolism of corals. Respiration in the light is difficult to quantify as most techniques measuring O_2_ flux only measure net rates. Following Edmunds & Davies [29], we therefore attempted to assess the effect of irradiance on respiration by measuring post-illumination respiration rate (*R*_L_). This was done by applying a 15 min dark treatment between each of the eight 15 min light treatments. The measurements of *R*_L_ were considered proxies for respiration of illuminated corals. Gross production for each irradiance step was subsequently calculated as the sum of *R*_L_ and net photosynthesis (both parameters normalized to symbiont cell density; figure 2). The calculated rates of gross photosynthesis enabled construction of photosynthesis versus irradiance (P–E) curves. The curves were fitted to a theoretical model of a hyperbolic tangent equation [23,30], whereby the descriptive parameters *P*_max_ (maximum gross photosynthetic rate), *α* (light use coefficient) and *E*_k_ (minimum saturating irradiance) were calculated. In addition to these calculations, the ratio between maximum gross photosynthesis rate and the dark respiration (P/R) was calculated for each specimen. In this instance, both parameters were normalized to symbiont cell density in order to estimate the production required to outweigh the energetic demand of the holobiont.

### (e) Physiological variables

Following the photobiological measurements, each coral sample was placed in a plastic bag with filtered (0.2 µm) sea water and air blasted until the tissue was removed from the skeleton. The resulting slurry was homogenized for 45 s using a tissue grinder and subdivided for symbiont density and chlorophyll *a* determinations.

Determinations of symbiont density were carried out using eight replicate counts in a Neubauer Improved Haemocytometer on a light microscope (Zeiss GmbH, Germany; bright field, optical compound microscope). Concentrations of chlorophyll *a* were determined with a double extraction in 100 per cent acetone. The optical properties of the extract were measured at 630 and 663 nm using a Shimadzu UV-1700 spectrophotometer (Shimadzu Corporation, Kyoto, Japan). All samples were stored at −20°C and in darkness during the extraction process. The concentration of chlorophyll *a* was determined using the formula from Jeffrey & Humphrey [31].

Total protein content was determined with the Petersons–Lowry total protein assay using bovine serum albumin as the standard for coral samples. Coral tissue was removed from the skeleton by stripping with an air gun in 100 mM phosphate-buffered saline with 5 mM beta-mercaptoethanol. To lyze cells, each sample was sonicated for 30 s and homogenized with a tissue grinder for 45 s. Each sample was then spun in a centrifuge at 3500*g* for 5 min at 4°C to separate cell debris from the solution. The supernatant was removed by pipette and stored at −80°C. Absorbance was measured at 750 nm on a spectrophotometer (SpectraMax, Molecular Devices, CA, USA). The average absorbance of duplicate samples was used to determine the protein content.

The surface area of *S. hystrix* was determined using wax dipping [32] and for *P. speciosa* by using image analysis software ([33], http://rsb.info.nih.gov/ij/). The physiological data were then normalized to the surface area of the sample.

### (f) Statistical analyses

The relationship between symbiont type and depth at South Scott Reef was first examined with a logistic regression to determine the significance of the changes in *Symbiodinium* genotypes observed in *S. hystrix* and *P. speciosa*. The influence of time, depth and symbiont type (independent variables) on the physiological response variables of *S. hystrix* and *P. speciosa* was then examined. Several models were developed for each physiological variable, including generalized linear regression and generalized additive models (GAMs). A log transformation was used for any physiological variables that were not normally distributed. Various measures of goodness of fit were applied to identify the best model, i.e. the best fit; these included *r*^2^, Akaike information criterion and Bayesian information criterion. Preliminary analyses showed that the output of the GAMs provided the best models for all physiological variables, which allow predictors to be fitted either as parametric or non-parametric smoothing terms (e.g. [34]). To test for overfitting, we increased the penalty on each model degree of freedom [35], and this had no effect on the model performance, i.e. demonstrating that overfitting did not occur. A Pearson correlation was used to further examine the relationship between symbiont types and the P/R ratio with changes in depth at South Scott Reef. All analyses were carried out using the statistical package R [36].

## 3. Results

### (a) Sea water temperature and irradiance

In November 2008, the mean sea water temperature decreased from 30.57 ± 0.03°C (*n* = 10) at 3 m to 28.23 ± 0.05°C (*n* = 4) at 50 m. By contrast, the water column was well mixed during April 2009 with sea water temperatures of 28.90 ± 0.01°C (*n* = 10) and 28.29 ± 0.05°C (*n* = 6) at 3 and 50 m, respectively. Mean midday scalar irradiance decreased exponentially with depth during both sampling events at South Scott Reef. The mean diffuse attenuation coefficient of scalar irradiance (*K*_d_ PAR) at the sampling sites at South Scott Reef was 0.0564 and 0.0584 m^−1^ in November 2008 and April 2009, respectively.

### (b) Symbiont type

Three distinct ITS1 SSCP profiles (1, 2 and 3) were observed in the *S. hystrix* samples, while three different profiles (4, 5 and 6) were encountered in the *P. speciosa* samples. None of the ITS1 sequences obtained from these samples are identical to *Symbiodinium* ITS1 sequences available in GenBank. Profile 1 consisted of two bands, which were cloned and sequenced (GenBank accession nos. HQ407536 and HQ407537). Both sequences showed highest similarity with a C-strain isolated from *Montipora* sp. from Magnetic Island in the central Great Barrier Reef (GBR) (GenBank accession no. EU567166) as well as with a few other closely related strains, i.e. 97 per cent and 99 per cent for the slowest and the fastest band in the SSCP gel, respectively. Profile 1 was observed in *S. hystrix* samples collected from depths greater than 24 m, with two exceptions being samples collected from 12 m depth. Profile 2 was uncommon and mostly found between 25 m and 32 m depth, with one exception of a sample collected between 10–12 m. Profile 2 consisted of a single band, which showed the same mobility in the SSCP gel as our reference sample for C1 (GenBank accession no. AF380551). However, its sequence (GenBank accession no. cHQ407538) showed only 98 per cent similarity to C1, and was equally similar to several other sequences in GenBank. It is likely that an intragenomic variant with equal SSCP mobility was picked up through cloning. Profile 3 was only found in samples collected from depths of less than 23 m. It consisted of four bands, but we only succeeded in cloning the fastest two (i.e. the bottom two bands in the SSCP gel), suggesting that the top two bands were SSCP artefacts. The fastest band (GenBank accession no. HQ407539) showed 99 per cent sequence similarity with a D-strain isolated from *Acropora millepora* from Magnetic island and Davies Reef in the central GBR (EU567171, EU567172, EU567174; [37]) and 98 per cent similarity with a range of sequences available for strain D1a (e.g. EU074900, EU074906). The slowest band (GenBank accession no. HQ407540) showed 99 per cent similarity with a range of D1a sequences (e.g. EU074906, EU074900, AF396629, AF396630). This depth-related pattern of *Symbiodinium* genotype distribution in *S. hystrix* was confirmed using ITS2 DGGE analyses for colonies collected in other parts of the Scott Reef system [38].

Ninety-four per cent of the *P. speciosa* samples showed the same *Symbiodinium* ITS1 SSCP profile (profile 4), spanning the full depth range sampled. This profile consisted of two bands; sequence analysis showed these bands (GenBank accession nos. HQ407541 and HQ407543) were 98–99% similar to *Symbiodinium* C3 (AF380554; *sensu* Van Oppen *et al.* [27]) as well as C-strains isolated from the coral *Plesiastrea versipora* (AY186569) and the foraminiferan *Amphisorus hemprichii* (e.g. EU828689, EU786049; [39]). Profile 5 was found in a single sample collected from 58 m depth. It consisted of four bands, but as with profile 3, we only succeeded in cloning the fastest two (i.e. the bottom two bands in the SSCP gel), suggesting that the top two bands were SSCP artefacts. These two bands (GenBank accession nos. HQ407543 and HQ407544) showed 98–99% sequence similarity with a range of D1a strains (e.g. EU567172, EU567174, EU074906). Profile 6 was also found in one sample only, collected from a depth of 10 m. This profile consisted of a single band (GenBank accession no. HQ407545), which showed 98 per cent sequence similarity to a C-strain isolated from *A. millepora* from Davies Reef, central GBR (GenBank accession no. EU567161), and a few others.

**Figure 3.**
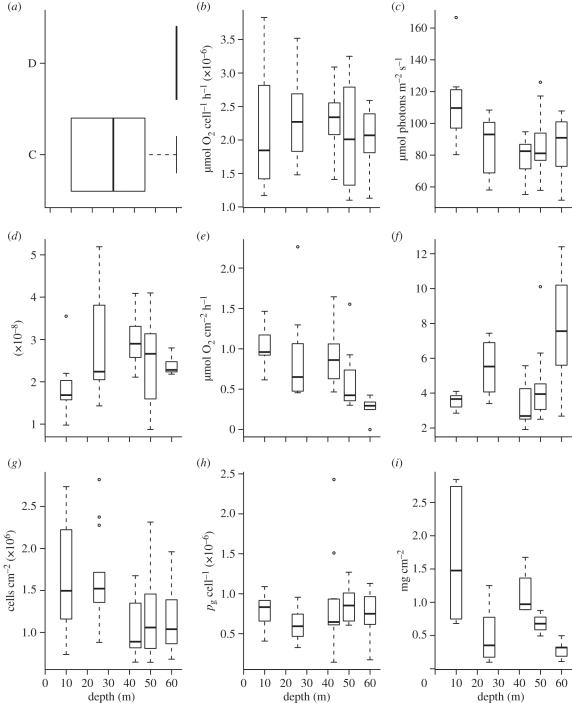
Box and whisker plots of the influence of depth on the physiological variables of *P. speciosa* at South Scott Reef. (*a*) Symbiont genotype; (*b*) *P*_max_; (*c*) *E*_k_; (*d*) *α*; (*e*) dark respiration; (*f*) P/R ratio; (*g*) symbiont density; (*h*) chlorophyll *a*/symbiont; and (*i*) total protein. For the lines in a box and whisker plot: error bars are the 95% confidence interval, the bottom and top of the box are the 25th and 75th percentiles, the line inside the box is the 50th percentile (median), and any outliers are shown as open circles.

**Figure 4.**
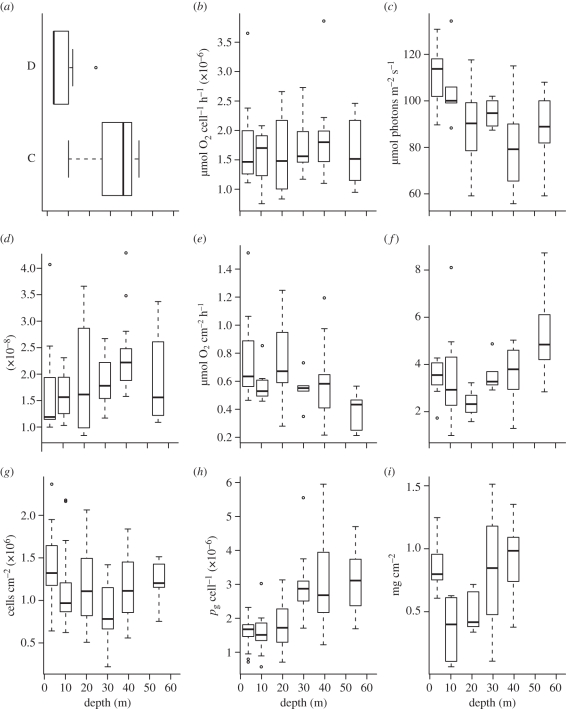
Box and whisker plots of the influence of depth on the physiological variables of *S. hystrix* at South Scott Reef. (*a*) Symbiont genotype; (*b*) *P*_max_; (*c*) *E*_k_; (*d*) *α*; (*e*) dark respiration; (*f*) P/R ratio; (*g*) symbiont density; (*h*) chlorophyll *a*/symbiont; and (*i*) total protein.

There were no significant differences in the distribution of symbiont genotypes over the depth gradient for *P. speciosa*, which harboured predominantly a single symbiont strain belonging to clade C from 10 to 60 m (figure 3). There were, however, significant differences with depth in the distribution of symbiont types associated within *S. hystrix*, where *Symbiodinium* D occurred between 3 and 23 m overlapping with a strain belonging to *Symbiodinium* C between 12 and 45 m (figure 4 and table 1). Depth accounted for approximately 71 per cent of the variance in *Symbiodinium* types associated with *S. hystrix*.

**Table 1.** Summary of logistic regression investigating the relationship between *Symbiodinium* type and depth for *P. speciosa* and *S. hystrix*. (Number in bold, *p* < 0.05.)

variate	slope	s.e.	*t*	*p*
*P. speciosa*				
symbiont type	25.90	17.45	1.48	0.149
*S. hystrix*				
symbiont type	−26.03	2.58	−10.11	**<0.001**

### (c) Photobiology

Patterns in the maximum rate of gross photosynthesis (*P*_max_) and the light use coefficient (*α*) for both species were dominated by great variability over the depth gradient (figures 3 and 4). By contrast, the minimum saturating irradiance (*E*_k_) of *P. speciosa* and *S. hystrix* at the shallowest depths was higher (27% and 21%, respectively) than the *E*_k_ of corals at the deepest depths (figures 3 and 4). The rate of dark respiration (*R*_D_) of *P. speciosa* and *S. hystrix* in the shallowest water was higher (58% and 22%, respectively) than that of corals at the deepest depths (figures 3 and 4). The ratio between the gross photosynthesis rate and dark respiration (P/R) of *P. speciosa* and *S. hystrix* at the deepest water was greater (34% and 37%, respectively) than corals in the shallowest depths (figures 3 and 4).

### (d) Physiological variables

The density of symbionts in *P. speciosa* and *S. hystrix* decreased with decreasing irradiance along the depth gradient at South Scott Reef. In *P. speciosa*, there were approximately 0.5–1.0 fold more symbionts per unit area within the shallowest corals than in corals in the deepest depths during both times of sampling (figure 3). This effect was less pronounced in *S. hystrix* with 10 per cent more symbionts per unit area within the shallowest corals versus the deepest depths during both times of sampling (figure 4). There was a doubling in the chlorophyll *a* per symbiont of *S. hystrix* as irradiance decreased from shallow to deeper water, but this pattern was less pronounced in *P. speciosa* (figures 3 and 4). Total protein per square centimetre of *P. speciosa* was greater (77%) in the shallowest corals. By contrast, total protein per square centimetre of *S. hystrix* showed a nonlinear response to depth, with colonies of this species having approximately 40 per cent lower levels of protein at 25 m than colonies at the shallowest and deepest depths (figures 3 and 4).

### (e) Influence of depth, time and symbiont type on physiological responses

**Figure 5.**
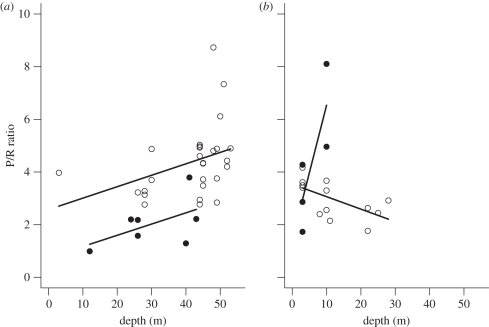
The influence of depth on the ratio between gross photosynthesis and dark respiration of *S. hystrix* for each symbiont type ((*a*) *Symbiodinium* C and (*b*) *Symbiodinium* D). Symbols: filled circles, November 2008; open circles, April 2009.

A summary of the GAMs to investigate the relationship between depth, time and symbiont type on the physiological response variables in each of *P. speciosa* and S. *hystrix* is provided in table 2. In general, the GAMs found that differences among depths and between times of sampling had a significant influence on the photobiological and physiological variables examined in both species. Interestingly, the GAMs showed that shifts in *Symbiodinium* types had an important influence on the ratio between the maximum gross photosynthesis and dark respiration (P/R ratio) of *P. speciosa* (*p* = 0.045) and *S. hystrix* (*p* < 0.001) (table 2). Moreover, the P/R ratio of *S. hystrix* varied inconsistently between time of sampling and symbiont type (time × symbiont type interaction, *p* = 0.002). For *Symbiodinium* C, the P/R ratio of *S. hystrix* increased with increasing depth at South Scott Reef during both times of sampling (positive correlations in table 3). For *Symbiodinium* D, however, the P/R ratio showed a positive response during November 2008 but a negative-linear response with increasing depth during April 2009 (table 3 and figure 5). By contrast, the P/R ratio of *P. speciosa* showed a positive relationship with increasing depth for both times of sampling.

**Table 2.** Summary of GAMs investigating the relationship between depth, time and symbiont type, and physiological response variables of *P. speciosa* and *S. hystrix. (p*-value and the *r*^2^ are shown for each model. Numbers in bold, *p* < 0.05.)

variate	depth	time	symbiont type	*r*^2^
*P. speciosa*				
*P*_max_ (µ mol O_2_ cell^−1^ h^−1^)	0.454	**0.014**	0.504	0.17
*E*_k_ (µ mol photons m^−2^ s^−1^)	**0.002**	**<0.001**	0.567	0.53
light use coefficient (*α*)	0.429	0.433	0.545	0.08
dark respiration (µ mol O_2_ cm^−2^ h^−1^)	**0.002**	**<0.001**	0.130	0.50
P/R ratio	0.158	0.544	**0.045**	0.12
symbiont density (cells cm^−2^)	**<0.001**	**<0.001**	0.172	0.42
chlorophyll *a*/symbiont (pg cell^−1^)	0.854	**<0.001**	0.532	0.25
total protein (mg cm^−2^)	**0.011**	—	0.863	0.28
*S. hystrix*				
*P*_max_ (µ mol O_2_ cell^−1^ h^−1^)	0.640	0.780	0.720	0
*E*_k_ (µmol photons m^−2^ s^−1^)	0.097	**0.016**	0.410	0.28
light use coefficient (*α*)	0.330	0.295	0.982	0.03
dark respiration (µmol O_2_ cm^−2^ h^−1^)	0.093	**0.024**	0.621	0.31
P/R ratio	**<0.001**	**<0.001**	**<0.001**	0.48
symbiont density (cells cm^−2^)	0.712	0.520	0.750	0.02
chlorophyll *a*/symbiont (pg cell^−1^)	**0.004**	**0.048**	0.631	0.36
total protein (mg cm^−2^)	**0.031**	—	0.776	0.23

**Table 3.** Summary of Pearson correlation analysis examining the relationship between P/R ratio and depth for each time and *Symbiodinium* type hosted by *S. hystrix*. (Numbers in bold, *p* < 0.05.)

	*r*^2^	*t*	d.f.	*p*
time 1, *Symbiodinium* C	0.77	2.41	4	**0.044**
time 1, *Symbiodinium* D	0.81	2.37	3	**0.049**
time 2, *Symbiodinium* C	0.36	1.94	25	**0.032**
time 2, *Symbiodinium* D	−0.61	−2.55	11	**0.014**

## 4. Discussion

Niche partitioning of specific *Symbiodinium* types driven by a differential use of irradiance is an important mechanism enabling a wide depth distribution for some corals. *Pachyseris speciosa* displayed a high affinity for the same *Symbiodinium* C types across its entire depth range (10–60 m), whereas *S. hystrix* displayed low symbiont specificity, with 93 per cent of shallow corals sampled harbouring *Symbiodinium* D over 3–23 m depth being replaced by corals harbouring *Symbiodinium* C in depths greater than 23 m to a maximal depth of 45 m.

The relationship between photosynthetic production and respiration of corals, also known as the P/R ratio, increased consistently with depth at both sampling times for *P. speciosa*, showing a reduction in metabolic energy consistent with the observed decrease in colony rates of respiration with depth. This pattern appeared the same for *S. hystrix* in November 2008, at the end of the southeast monsoon (i.e. Austral winter). However, in April 2009, at the end of the northwest monsoon (i.e. Austral summer), shallow colonies of *S. hystrix* exhibited decreased P/R ratios down to a depth of approximately 23 m, below which the ratio increased towards the maximum depth sampled at 55 m. Results of the statistical analysis showed that there was a significant relationship between the P/R ratio and symbiont genotype. Indeed, the change in direction of the P/R ratio correlated with a complete shift from *Symbiodinium* D- to C-dominated *S. hystrix* colonies at 23 m depth. It should be noted that while no separation of the contribution of respiration was made here between host and symbionts, a complete understanding of the mechanism associated with the changes in respiration with depth has not been possible. Nevertheless, our data indicate a physiological niche specialization among *S. hystrix* colonies, where those containing *Symbiodinium* D had a competitive advantage in high-irradiance habitats, i.e. shallow-water, over those colonies containing *Symbiodinium* C. This advantage, however, comes at a metabolic cost with increased depth as evidenced by a reduction in the P/R ratio in April 2009 following increased irradiances during the Austral summer. These results add to the growing body of evidence that symbiont genotype can influence holobiont energetics and productivity [17,19].

Scott Reef suffered a severe bleaching event in 1998 (up to 80% relative decrease in hard coral cover; [40]), which may have driven some short-term shifts in symbiotic affinities. Such large-scale bleaching has been suggested to result in shuffling of symbionts from thermo-sensitive (*Symbiodinium* C2) to thermo-tolerant (*Symbiodinium* D) types in the coral *Acropora millepora* on the GBR [41,42]. However, the observed depth distribution pattern of *Symbiodinium* within *S. hystrix* in the present study is most probably owing to photo-acclimatization, given that recent evidence suggests that corals revert to their original *Symbiodinium* consortium within 1–2 years after severe thermal disturbance provided that environmental conditions are comparable [42,43]. In the absence of disturbances, coral–algal symbioses show high levels of temporal stability [1,43,44]. Indeed, Stat *et al*. [45] observed that *S. hystrix* from the GBR maintained an association with the same *Symbiodinium* strain over a 1 year period that included a thermal-stress event, reinforcing the notion that changes in the symbiont types on small time scales owing to environmental perturbations is not a universal characteristic of reef-building corals. Nevertheless, bleaching stress responses of corals are typically induced by high irradiance and temperature in combination (e.g. [46–48]) and are therefore likely to be more frequent in shallow than in deep habitats.

The adoption by *S. hystrix* of a different *Symbiodinium* type in the shallows is likely to confer greater resilience to environmental extremes. The nonlinear protein content response observed for *S. hystrix*, which corresponded with the change in *Symbiodinium* distribution along the depth gradient, lends support to this view. While associated with *Symbiodinium* C, *S. hystrix* showed a decrease in protein content at shallow depths, which along with a decreasing P/R ratio indicates an increase in metabolic cost of hosting *Symbiodinium* C when exposed to higher irradiances. A response in protein content to environmental change is further supported by Fitt & Cook [49] who found that shaded polyps in a hydroid had reduced tissue biomass (i.e. protein content) compared with unshaded polyps. By contrast, an increase in protein content was observed towards the surface for *Symbiodinium* D-containing conspecifics, promoting the suggestion that the shift to this association confers higher resilience to shallow-water conditions and is particularly photophilic. *Pachyseris speciosa* maintained higher protein content in shallow than in deep conspecifics, suggesting that its association with *Symbiodinium* C across the entire depth gradient is metabolically sufficient and suitable to high- as well as low-light conditions.

While evidence of *Symbiodinium* depth-zonation patterns in mesophotic communities has been reported previously (e.g. [7,22]), this study is, to our knowledge, the first to link direct metabolic flux measurements as well as tissue biomass with symbiont types over a large depth gradient incorporating mesophotic depths. These results suggest that photobiological flexibility of both host and symbionts is important to cope with contrasting light regimes and to maintain strong functional links between shallow and mesophotic coral communities. In addition, our results demonstrate that although shifts in *Symbiodinium* types with depth may confer a competitive advantage that allows some corals to colonize specific light habitats over a large depth gradient, such shifts may incur a metabolic cost highlighting the trade-off between niche specialization and diversification.
